# Benefit of Asian pigmented rice bioactive compound and its implication in breast cancer: a systematic review

**DOI:** 10.12688/f1000research.130329.1

**Published:** 2023-04-05

**Authors:** Wirdatun Nafisah, Alexander Patera Nugraha, Aditya Nugroho, Andi Isti Sakinah, Duano Sapta Nusantara, John Philia, Mohammad Iqbal Kurniawinata, Wirdatul Aini, Vika Tresnadiana Herlina, Tengku Natasha Eleena binti Tengku Ahmad Noor

**Affiliations:** 1 Department of Biology, Faculty of Mathematics and Natural Sciences, Universitas Brawijaya, Malang, East Java, 65145, Indonesia; 2 Department of Orthodontics, , Faculty of Dental Medicine, Universitas Airlangga, Surabaya, East Java, 60132, Indonesia; 3 Department of Silviculture, Faculty of Forestry and Environment, Institut Pertanian Bogor, Bogor, West Java, 16680, Indonesia; 4 Department of Agriculture, Universitas Hasanuddin, Makassar, South Sulawesi, 90245, Indonesia; 5 Department of Mathematics Education, Faculty of Teacher Training and Education, Universitas Sriwijaya, Palembang, South Sumatra, 30139, Indonesia; 6 Department of Mechanical Engineering, Faculty of Engineering, Universitas Diponegoro, Semarang, Central Java, 50275, Indonesia; 7 Department of Aquaculture, Faculty of Fisheries and Marine Science, Institut Pertanian Bogor, Bogor, West Java, 16680, Indonesia; 8 Department of Mathematics, Faculty of Mathematics and Natural Sciences, Institut Teknologi Bandung, Bandung, West Java, 40132, Indonesia; 9 Department of Food Science and Techology, Faculty of Agricultural Engineering, Institut Pertanian Bogor, Bogor, West Java, 16680, Indonesia; 10 Dental Clinic, Angkatan Tentera Malaysia, Kuching, Malaysia

**Keywords:** medicine, cancer, non-communicable disease, rice, food

## Abstract

**Background:** Utilizing the bioactive compounds found in pigmented rice might significantly reduce the risk of breast cancer. This study aims to systematically review existing literature on the benefit of Asian pigmented rice bioactive compounds and their implication in breast cancer.

**Methods:** Searches of the literature were conducted in two databases (Scopus and PubMed) for a systematic review. The keywords resulted in a total of 407 articles, consisting of 103 PubMed and 304 Scopus articles. 32 manuscripts were excluded because the article was over 10 years old. After excluding book chapters and non-English languages, we had 278 potential articles to be reviewed. After checking and screening the title and abstract and eliminating duplicate articles, then 66 articles were obtained. After the selection and elimination of the full-text manuscripts, finally 10 of them which met the inclusion criteria.

**Result:** The included studies in this review were entirely based in Asia. The year of publication ranged from 2013 to 2020. Half of included studies used black rice extract, two used red jasmine rice extracts, and three used Korean rice extracts (black, red, dark purple and brown rice). All studies were conducted *in vitro* and three studies were compared with *in vivo* tests on female mice. The pigmented rice is mainly black, red, and dark purple rice, and contains a variety of peonidin-3-glucoside, cyanidin-3-glucoside, γ-oryzanol, γ-tocotrienol, proanthocyanidin, cinnamic acid, and anthocyanins that may act as pro-apoptotic, anti-proliferative, and anti-metastasis of the breast cancer cells.

**Conclusion:** Pigmented rice is a beneficial food which possessed bioactive compounds that may have significant potential concerning a breast cancer.

## Introduction

Most deaths throughout the world are now caused by non-communicable diseases (NCDs). ^1^ The recognition of the challenges of NCDs has become a global spotlight as stated in the sustainable development goals (SDGs) on the target 3.4 by 2030, reduce by one-third pre-mature mortality from NCDs through prevention and treatment, and promote mental health and wellbeing. ^2^ Cancer is one of NCDs that has become one of the leading causes of death worldwide with an estimate of 10.0 million deaths in 2020. ^3^

An estimated 10.0 million people will die from cancer, a multifactorial illness, in the globe in 2020. ^4^ Breast cancer is the most diagnosed cancer type found in women and poses a serious public health concern globally. ^5^ An estimated breast cancer occurrence is 2.089 million in 2018 which is reported to increase continuously in all countries of the world. ^3^ There are major risk factors for breast cancer, such as aging, family history, reproductive factors, and life style. ^6^ Some of the factors are beyond individual controls and some are not. The factors that are within an individual’s control that can be altered to minimize the chance of getting breast cancer, include diet and lifestyle. ^7^ Different food patterns may be one of the most fundamental factors for this. ^8^ Rice is a staple food of dietary calories for half of humanity and has been widely demonstrated as a chemopreventive component. ^9^ Whole-grain pigmented rice, in contrast to white rice, contains a higher concentration of bioactive phytochemicals with various health benefits. ^8^

Due to the high levels of polyphenol and anthocyanins in colored rice, it is regarded as a functional food in Asia. ^10^ Many varieties of rice include colorful pigments, which are often concentrated in the pericarp or the bran of the rice kernel and give the grain hues like brown, red, purple, and black. ^11^ The flavonoids anthocyanin and proanthocyanidin, which are recognized to have nutritional benefits, are responsible for these color changes. However, their efficacy varies depending on rice color or variety. ^12^^,^^13^

The presence of polyphenols, which have been shown to have antioxidant, anti-inflammatory, and anti-adipogenic potential in *in vitro* and *in vivo* investigations, ^8^^,^^14^ has been predominantly cited as the cause of the possible health advantages of this type of rice. Black, red, and brown rice bran's phenolic and flavonoid components, as well as their free and bound fractions, antioxidant and anti-proliferative properties, have all been documented. ^13^^,^^15^^–^^17^

Thus, the aim of this study is to systematically review existing literature on the benefit of Asian bioactive compounds within pigmented rice and their implication in breast cancer.

## Method

### Search strategy

Articles for the current review were acquired from two electronic databases (PubMed and Scopus), considering that they hold the most articles for biomedical and pharmaceutical studies. The database literature search was conducted from November 8 to November 10, 2022. We created the keywords for each database. For PubMed, we used the following keywords; (((((cancer) OR (“breast cancer”)) OR (metastasis)) AND ((((((rice) OR (“pigmented rice”)) OR (“black rice”)) OR (“red rice”)) OR (“purple rice”)) OR (“brown ice”)) AND ((“cancer therapy”) OR (“anti-metastasis”)). Meanwhile for Scopus, the keywords were "Cancer" OR metastasis OR “MCF-7” OR “Breast cancer” OR tumor AND “Pigmented Rice” OR rice OR paddy OR “Oryza sativa” AND “Cancer Therapy” OR medicine OR pharmaceutical OR physicochemical. During literature search, only free access articles were used.

### Inclusion criteria

All types of experimental and observational studies written in English language were included. All duplicate studies and literature reviews were excluded from the final selection. Study subjects focused on breast cancer, and any other objects of *in vivo* and *in vitro* studies. Study factors included in the studies were the types of pigmented rice, other interventions in the form of extracts, whole grain, bran and hull. The research results include cancer therapy, pharmaceutical, and bioactive compounds as anti-cancer.

Study selection, data extraction, and quality assessment

The aforementioned keyword search resulted in a total of 407 articles, consisting 103 and 304 articles from PubMed and Scopus, respectively. We excluded 32 manuscripts because the article were published more than 10 years ago. After excluding book chapters and papers not in English, we had 278 potential articles to be reviewed. After checking and screening carefully the title and abstract of the manuscript, the duplicate articles were eliminated, then 66 articles were obtained. The authors reviewed the full text of those studies and finally selected 10 of them which met the inclusion criteria. 46 articles did not meet the criteria because duplicated, a book chapter, not accessible, not in English and no full-text. The flow diagram of the selection of study is shown in. ^26^^,^^27^

## Results

### Characteristics of the articles

The included studies in this review were entirely of Asian. The year of publication ranged from 2013 to 2020. Five studies used black rice extract, two used red jasmine rice extracts, and three used a mixture of Korean rice extracts (black, red and brown rice). All studies were conducted *in vitro* and three studies were compared with *in vivo* tests on female mice. The summary of the included study were: tabulated based on the results, active compounds and sample breast cancer cell lines which are presented in Table 1.

**Table 1.** The summary of studies discovered benefit of Asian pigmented rice physicochemical properties and its implication in breast cancer. ^29^

Author, year	Derived from	Compound	Samples	Settings	Findings	Conclusions
Liu *et al.*, 2013	Black rice ethanolic extract using supercritical CO _2_ extraction	Peonidine-3-glucoside and cyanidin-3-glucoside	Human breast cancer cell lines and mice breast cancer model	*In vitro* (BT474, MDA-MB-453, HCC1569, MCF-7, SUM190, and MDA-MB-231) *In vivo* (female nude mice)	A significant decrease of cell proliferation *in vitro* and tumor size *in vivo.* Anti-proliferative activity was mediated by the inhibition of phospho-HER2 phospho-AKT phopspho-p44/42MAPK, and apoptosis induction via caspase 3/7.	Peonidine-3-glucoside and cyanidin-3-glucoside compounds posed and potential as anti-proliferative and pro-apoptotic in breast cancer therapy.
Luo *et al.*, 2014	Black rice extract	BRA-90 anthocyanins (BRACs)	Human breast cancer cell lines	*In vitro* (MCF7, MDA-MB-453 and MCF10A) *In vivo* (female BALB/c mice)	In a concentration-dependent way, BRACs reduced the activity of urokinase-type plasminogen activator, a factor that promotes transfer, and they also inhibited migration, adhesion, motility, and invasion (u-PA). In addition, BRACs also diminished lung tumor nodules in mice and blocked pulmonary metastasis and tumor development.	Through the suppression of molecules that promote metastasis, BRAC has anti-metastatic potential against human breast cancer cells that are ErbB2-positive.
Chung *et al.,* 2015	Methanolic extract of three Korean cultivars rice; ilpum, Heugjinju, and Jeogjinju. And one japonica weedly rice cultivars; WD-3	3-Phenylpropanoic acid, 3,4-Dihydroxybenzoic acid, 2-Propenoic acid, 3-phenyl-, methyl ester, 2-Propenoic acid, 3-(4-hydrocxyphenyl)-, methyl ester, 2-Prepenoic acid, 3-(4-hydroxyphenyl)-, (Z)-, 2-Propenoic acid, 3-(3-hyroxyphenyl)-, methyl ester, Cinnamic acid [(2E)-3-Phenylacrylic acid, Hexadecanoicacid	Human breast cancer cell lines	*In vitro* (MCF-7)	The WD-3 hull extract decreased cell growth and induced G0/G1 phase arrest by inhibiting cyclins and cyclin-dependent kinases. Inhibition of p21 expression decreased the G1 phase arrest induced by WD-3 extract. Cinnamic acid derivatives were found to be the primary active ingredients in the F4 fractioned from WD-3.	The most phenolic substance was found in the pigmented wild rice hulls (WD-3). The primary ingredients that assist growth inhibition and cell cycle arrest in breast cancer cells are cinnamic acid and its derivatives.
Pintha *et al,* 2014	Ethanolic extract of red jasmine rice and its fraction (n-hexane, dichloromethane, and ethyl acetate	Proanthocyanidin, γ-oryzanol, γ-tocotrienol, γ-tocopherol	Human breast cancer lines	*In vitro* (HT1080 and MDA-MB-231)	Ethanolic ectract of red jasmine rice (ECC) and its fraction (hexane (Hex) and dichloromethane (DCM)) were able to inhibit HT1080 and MDA-MB-231 cancer cell invasion. However, the highest potency showed by Hex and DCM than ECC. Moreover, ethyl acetate fraction had no effect.	The anti-invasion potential of red jasmine rice extract caused by the activity of the bioactive content through the inhibition of MMPs secretion and activity.
Pintha *et al.,* 2015	Ethanolic extract of red jasmine rice and its fraction (n-hexane, dichloromethane, and ethyl acetate	Proanthocyanidin	Human breast cancer cell lines	*In vitro* (MDA-MB-231)	Through the inhibition of ECM degradation-associated proteins like matrix metalloproteinase-9 (MMP-9), membrane type-1 matrix metalloproteinase, urokinase plasminogen activator, urokinase plasminogen activator receptor, and plasminogen activator-1, proanthocyanidin rich fraction from red rice (PRFR) inhibited MDA-MB-231 invasion. Additionally, it significantly reduced the release of intercellular adhesion molecule-1 and interleukin-6, as well as collagenase and MMP9 activities.	Proanthocyanidin from red rice (PRFR) inhibited the invasion of MDA-MB-231 breast cancer cells via modulating the expression of invasion-associated proteins, presumably by inhibiting NF-κB activity.
Baek *et al.,* 2015	Methanolic extract of three Korean cultivars; Ilpum, Heungjinju, Jeogjinju, and one Japonica weedy rice; WD-3	Phenolic Flavonoid	Human breast cancer cell lines	*In vitro* (MCF-7, B16F10, M-3, and YD-38 cell)	Pigmented rice bran and hull extracts had significantly higher levels of phenolic and flavonoidic compounds, DPPH radical scavenging activities, and reducing abilities than Ilpum extracts. In human breast, melanoma, and oral cancer cells, hull extracts shown more cytotoxicity than bran extracts, with Heugjinju hull extract being the most powerful. Cellular shrinkage, DNA breakage, and nuclear condensation suggest that hull extract-mediated.	Hull extracts showed greater potential than bran extracts in terms of cytotoxicity in human breast, melanoma, and oral cancer cells, where Heugjinju hull extract being the most potent.
Chen *et al.,* 2015	Black rice extract	Anthocyanins (BRACs)	Human breast cancer cell lines	*In vitro* (MDA-MB-453)	BRACs inhibited the migration and invasiveness of MDA-MB-453 HER+ cells through RAF/MAPK pathway showed by the inhibitory effect of BRACs against siRNA-mediated RAF/MEK/ERK pathway. In addition, BRACs also reduced the expression of metastatic related protein such as raf, mek, and jnk in MDA-MB-453 cells. This extract aslo tend to suppress the phosphorilation of RAF and MAPKs protein. The antimetastatic effect of BRACs was mediated by the ability of this extract on the inhibition of MMP2 and MMP9 expression.	BRACs suppressed the migration and invasion HER2+ breast cancer (MDA-MB-453) through the inhibition of RAF/MAPK pathway. Besides, BRACs also showed anti-metastatic effect mediated by the inhibition of MMP2 and MMP9 protein expression.
Zhou *et al.,* 2017	Black rice extract	Anthocyanins (BRACs)	Human breast cancer cell lines	*In vitro* (MCF10A, MCF7, and MDA-MB-453)	BRACs inhibited the invasive ability of HER-2-positive human breast cancer cells, resulting in a 68% reduction in the number of invaded cells. They also reduced the HER-2-positive human breast's migration distance from cancer cells by 37% when compared to the control cells group that was not treated.	Metastasis suppression by BRACs mediated through cSrc/FAK/p130Cas pathway which is one the most pivotal pathway in breast cancer.
Ghasemzadeh *et al.,* 2018	Methanolic extract of black, red, and brown rice bran	Cinnamic acid, *p-*coumaric acid, catechin, myrecetin, and quercetin.	Human breast cancer cell lines	*In vitro* (MCF-7, MDA-MB-231, and MDA-MB-453)	Different coloured rice brans varied significantly in their phytochemical contents and biological activity. Black rice bran has the greatest phytochemical concentration, followed by red and brown rice bran. Except for ferulic acid and p-coumaric acid, the concentration of the free phenolic and flavonoid compounds was much greater than that of the bound compounds. Black rice bran extracts showed the highest antioxidant activity, followed by red and brown rice bran extracts. With half maximal inhibitory concentrations (IC50) of 148.6 and 119.2 mg/mL against MCF-7 and MDA-MB-231 cell lines, respectively, extracts of black rice bran demonstrated strong antiproliferative activity in contrast to the activity of the extracts of red rice bran (175.0 and 151.0 mg/mL, respectively) and brown rice bran (382.3 and 346.1 mg/mL, respectively).	The highest antioxidant and anti-proliferative activity was black rice extract followed by red and brown rice.
Teng *et al.,* 2020	Black rice extract	Anthocyanins (BRACs)	Murine breast cancer cell lines	*In vitro* (4T1) *In vivo* (female BALB/c)	BRE contained 25% anthocyanins inhibited the metastasis of 4T1 cancer cell lines by suppression EMT through the reduction of snail, vimentin, and E-cadherin expression. The *in vivo* study showed that this extract has no obvious systemic toxicity on the mice.	BRE able to suppress EMT and metastasis through the inhibition of metastasis related molecules. This extract has low toxicity and cause no obvious systemic toxicity.

Methodological quality

We reviewed results from 10 studies, seven of which involved *in vitro* experiment and three of which involved both *in vitro* and *in vivo* experiment. Unfortunately, there are no specific studies that focus on *in vivo* experiments. In detail, we report 10 samples from *in vitro* studies and three samples from *in vivo* studies.

*In vitro*

Many different cells have been used in *in vitro* experiments, including from human and murine breast cancer. ^13^^,^^18^^–^^21^ Besides, there are several single-cell *in vitro* studies, among which are subcutaneous MDA-MB-453 cells, ^18^^,^^19^ MCF-7 cells, ^13^ MDA-MB-231 cells, ^20^ and 4T1 cells. ^21^ Furthermore, several studies used multiple cells, such as, ^15^ which used HER2-positive (BT474, MDA-MB-453, and HCC1569 cells) and HER2-negative (MCF-7, SUM190, and MDA-MB- 231) breast cancer cell lines. The HT1080 fibrosarcoma and MDA-MB-231 cell lines were utilized in. ^22^^,^^23^ The cells used by ^16^ were MCF-7, B16F10, M-3, and YD-38. The cells used by ^24^ were MCF-10A, MCF-7, and MDA-MB-453. In summary, the classification of breast cancer cells used in the 10 articles analyzed in this study were human triple-negative breast cancer (MDA-MB-231), human ERα-positive breast cancer (MCF-7), human HER2-positive breast cancer (BT474, MDA-MB-453, and HCC1569 cells), human HER2-negative (MCF-7, SUM190, and MDA-MB- 231), and murine breast cancer (4T1 and M3). There were also non breast cancer cell line used in this study, such as fibrosarcoma cell lines (HT1080), murine melanoma cell lines (B16F10), and human Squamous cell carcinoma cell lines (YD-38).

*In vivo*

Experiments *in vivo* using same species of mouse, naked female mice, were carried out with different cancer cells. Two studies employed 4-week-old mice, ^18^^,^^21^ while one used 6-7-week-old mice. ^15^ In these experiments, several cells types were used and injected into the flank of mammary fat pad of mice to construct the breast cancer mice model. HER2-positive MDA-MB-453 cells were employed in one study. ^15^ Besides, the previous study, ^18^ found MDA-MB-453 (ErbB2-positive) was employed alongside MCF-7 (ErbB2-negative) and MCF-10A (normal) cells. In the last study, 4T1 cells were used to be transplanted to the mice. ^21^

### The benefit of Asian pigmented rice bioactive compound

Pigmented rice cultivars differ in terms of both qualitative and quantitative characteristics. ^10^^–^^12^ This rice variety is distinguished by its mostly red, black, or purple pericarps. Wide-color rice varieties have drawn more attention because of their many biological functions. The pigments serve as sources of phytochemicals when contrasting the nutritional value of colored rice with white rice brans. Anthocyanidins, ferulic acid, diferulates, anthocyanins, and polymeric proanthocyanidins are some of the phenolic chemicals. ^10^^,^^11^

Pigmented rice residues are attractive sources of bioactive substances such as phenolic antioxidants and anticancer agents for the benefit of human health. ^12^^,^^13^ Anthocyanin is found in the bran layers of the rice kernel, whereas phenolic acids are primarily present in the bran layers of rice in their free, conjugated, and bound forms. ^16^ The two primary bioactive phenolic chemicals found in cereal grains, mostly found in the pericarp of colored types, are anthocyanins and proanthocyanidins. ^14^ Anthocyanins, which are primary metabolites found in the bran layers of rice kernels, have been identified as functional dietary components that promote good health and have anticancer, antioxidant, hypoglycemic, and anti-inflammatory activities. ^11^

Considering the numerous health benefits connected with functional components, such as anti-inflammatory, antioxidative, and anticancer properties, pigmented rice is considered an available food and food ingredient in many Asian countries. ^10^^–^^14^ The anticancer effect is suggested partly through the enhancement of bioactive substances such as vitamin E, phytic acid, γ-aminobutyric acid (GABA), γ-oryzanol, and phenolics. ^9^

Black rice

A traditional and healthy cuisine, black rice is mostly grown in East Asia. Phytochemicals, tocopherols, polyphenols, B vitamins, and anthocyanins are only a few examples of the water-soluble bioactive substances abundant in black rice. ^21^ These substances might help your health and shield you against long-term conditions linked to oxidative stress and antioxidant activity. ^10^ A significant amount of phytochemicals are present in black rice bran, giving it strong medicinal properties. Black rice extract anthocyanins dramatically decreased the metastasis of breast cancer, according to seven research that were considered. The aleurone layer of black rice is where black rice anthocyanins (BRACs) are found. Human breast cancer cells that are ErbB2-positive can be prevented from metastasizing by BRAC. ^18^ Additionally, Liu *et al.* (2013) demonstrated that black rice extract includes peonidin-3-glucoside and cyanidin-3-glucoside, which can diminish the tumor size and volume. ^15^

Red rice

Two studies found that red jasmine rice, which contains proanthocyanidins, inhibits breast cancer cell proliferation. Most of the phenolic chemicals in red rice, which are responsible for the red coloring of the pericarp, are proanthocyanidins and catechins. ^20^^,^^22^ Proanthocyanidin from red rice (PRFR) contains procyanidins and prodelphinidins. ^22^ Moreover, red rice extract also has a high concentration of γ-oryzanol and γ-tocotrienol in the Hex and DCM fractions. ^20^

Purple rice

A variety of rice varieties, including glutinous rice, are referred to as purple rice and have historically been eaten throughout Eastern and Southeast Asia. ^23^ The pigments are naturally occurring compounds belonging to the flavonoid family in which cyanidin, pelargonidin, peonidin, delphinidin, petunidin, and malvidin represent the most commonly occurring anthocyanin aglycone. ^11^ One study found that purple rice hulls contained the highest phenolic compound. ^23^ Cinnamic acid and its derivatives are the primary ingredients for breast cancer cell growth suppression and cell cycle arrest. ^13^

### Implication of bioactive compound in breast cancer

Phenolic and flavonoid chemicals act as an antioxidant to protect cells from oxidative damage, which is a common cause of aggressive diseases like cancer. ^16^ However, the anticancer activities were not directly correlated with total phenolic or total flavonoid content values. ^16^ So, phenolic and flavonoid from many types of pigmented rice have also been tested for apoptotic activity revealing that cell shrinkage and nuclear condensation occur in human breast cancer cell line MCF-7. ^16^ The antioxidant activity of rice phenolic compounds is detectable in parallel with the inhibition of proliferation of MCF-7 cells through G1 cell cycle arrest and comparable to quercetin as an antioxidant standart. ^13^

Proanthocyanidins are monomeric flavan-3-ol oligo- or polymers that are created as a byproduct of the flavonoid biosynthesis process. ^20^ Proanthocyanidins, which are essentially concentrated tannins, are made by cytosolic multienzyme complexes acting along the phenylpropanoid route. ^22^ By incorporating sugars, anthocyanidins are transformed into anthocyanins. ^19^ Proanthocyanidins turn into anthocyanins when heated in an acidic medium. As byproducts of the flavonoid pathway, proanthocyanidins and anthocyanins have the same metabolic intermediates. Proanthocyanidins, in the form of procyanidin (catechin and/or epicatechin) and prodelphinidin (epigallocatechin and/or gallocatechin) showed anti-metastatic effects in cancer breast MDA-MB-231 and HT1080 cells. ^22^ It reduces the cell invasion and migration in a dose dependent manner with IC _50_ at 7.52±1.42 μg/mL and 10.6±0.59 μg/mL respectively and as a molecular mechanism of metastasis it inhibits collagenase activity. ^20^ Suppression of breast cancer metastasis including inhibition the mRNA expression of their signaling pathway has also been known. ^19^

The anthocyanins pigment such as cyanin-3-glucosides or peonidin-3-glucosides known to dose-dependent inhibition of cell proliferation and suppress the tumour growth in HER2-positive cancer lines which associated with breast cancer. ^15^ Inherent with the previously studied that both compounds inhibit cell human ductal breast carcinoma HS578T growth by blocking cell proliferation at the G(2)/M phase so that it cannot form a normal mitotic apparatus, lowering some cancer response proteins, and chromatin condensation leads to cell death. ^23^ Anti-metastasis action prevents cancer cells from invading the blood or lymphatic vessel and spreading to other tissues or organs, among other benefits. The anthocyanins found in black rice are able to lower almost 40% of tumour nodules with *in vivo* studies and about 10% inhibits invasion of cancer breast MDA-MB-453 cells. ^18^ Aprevious study reported a higher invasive inhibition of cells by 68% and also a decrease in the migration distance of MDA-MB-453 cells by 37% with anthocyanins compared to untreated cells after 24 hours. ^24^

### Strengths and limitations of the review

Several bioactive substances with the potential to operate as anti-metastatic agents, including anthocyanins, phenolics, and flavonoids found in different kinds of pigmented rice, are among the articles employed. To ensure the efficacy of each bioactive chemical, however, more *in vivo* research or randomized clinical trials with multicenter investigations are required as the majority of them are *in vitro* studies.

### Significance of the findings and possible mechanism

**Figure 1.**
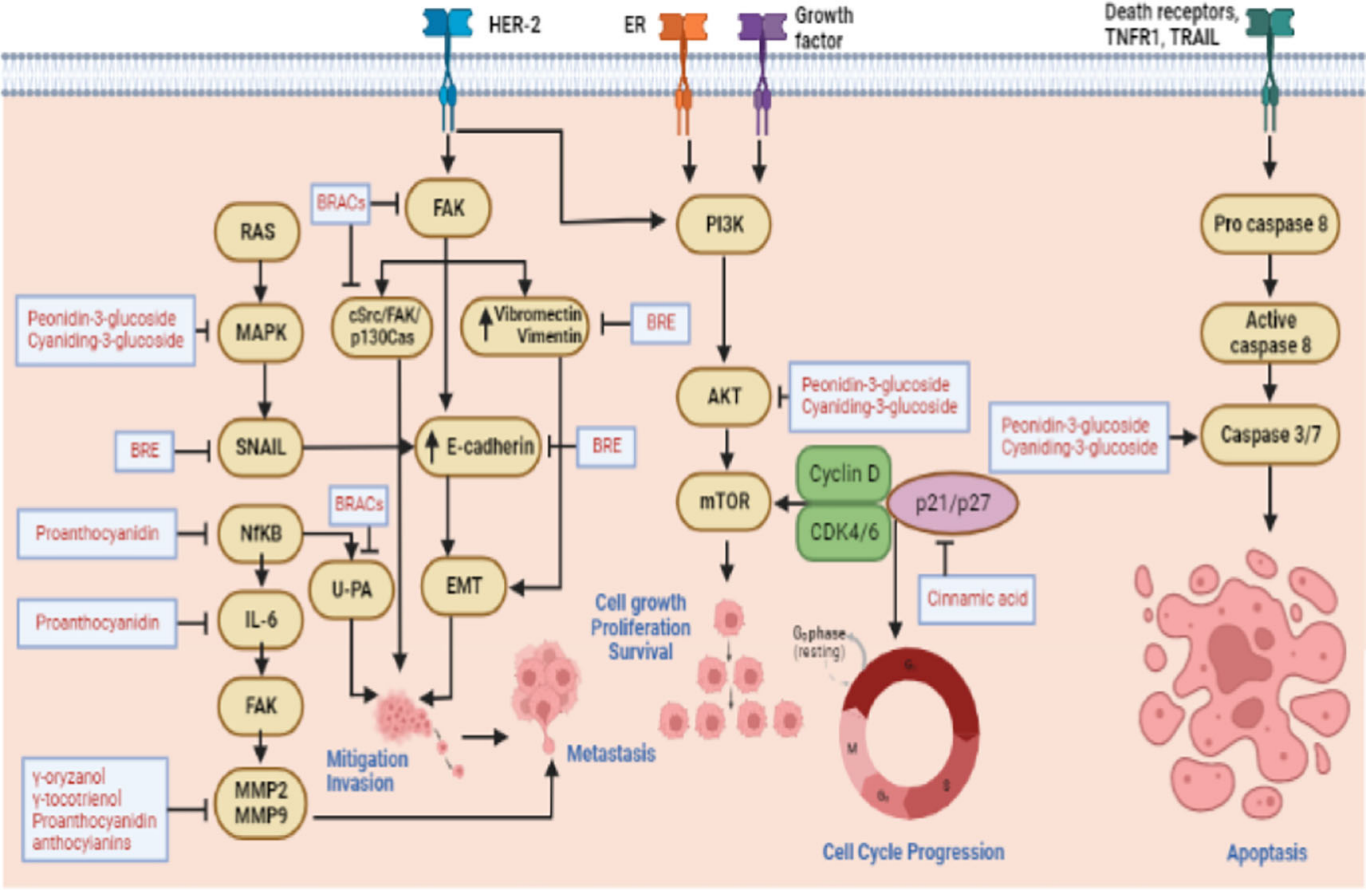
Pigmented rice bioactive compound possible mechanism from breast cancer. ^28^

Breast cancer is a complex and multifactorial disease that involves many cellular and molecular pathways. Weinberg and Hanahan in 2000 stated 6 hallmarks of cancer, (1) continuous proliferative signaling, (2) escaping growth suppression, (3) countering cell death, (4) sustaining replicative immortality, (5) promoting angiogenesis, and (6) metastasis. ^25^ To overcome this complex phenomenon, an effective therapy needs to be found. Due to the multi-target cancer therapy, natural product consumption is currently a major problem. As a result, this study investigated the bioactive chemicals found in Asian pigmented rice that were intended to target many pathways in the treatment of cancer ( Figure 1).

This study found that bioactive compounds from the 10 articles reviewed, involved in several pathways on breast cancer, such as metastasis, cell growth and proliferative signaling, cell cycle progression and apoptosis. ^13^^,^^15^^–^^24^

Black rice extract with unidentified compounds has anti-metastatis activity through the reduction of anti-metastasis related protein expression such as snail, vimentin, and E-cadherin. ^21^ Besides, peonidin-3-glucoside, cyaniding-3-glucoside from black rice, and cinnamic acid from WD-3 varieties of Korean rice showed anti-proliferative activity on breast cancer. ^15^ Peonidin-3-glucoside and cyaniding-3-glucoside of black rice also promoted apoptotic activity through the induction of caspase 3/7. ^15^^,^^16^ Anti-metastatic pathway was mostly found to be involved in the implication of pigmented rice bioactive compounds against breast cancer. ^22^ This study found that γ-oryzanol, γ-tocotrienol, and proanthocyanidin from red rice reduced MMP2 and -9 secretion. ^22^ In addition, MMP9 along with ICAM, IL-6, and NFkB were reduced after treated by red jasmine rice that contained proanthocyanidin. ^20^ Anti-metastasis also mediated by black rice anthocyanins through the reduction of FAK, cSrc and p130Cas phosphorylation. Black rice anthocyanins also posed anti-metastatic activity through the suppression RAF/MEK/ERK pathway and MMP2,-9 downregulation. ^19^ Lastly, black rice anthocyanins can suppressed u-PA involved in tumor invasion. ^18^

## Conclusions

The pigmented rice covered by this study were black, red, and dark purple rice, and all contains a variety of peonidin-3-glucoside, cyanidin-3-glucoside, γ-oryzanol, γ-tocotrienol, proanthocyanidin, cinnamic acid, and anthocyanins that may act as pro-apoptotic, anti-proliferative, and anti-metastasis in breast cancer cells. Therefore, choosing whole-grain red, black, or purple is an excellent choice for health. Plus, these varieties are richer in disease-fighting antioxidants. Rice being a staple food for half of the world can be a source of energy for our generations only if it is accumulated with nutrition.

## Data Availability

No data associated with article.

## Reporting guidelines

Figshare: PRISMA_2020_checklist. Pigmented Rice Bioactive Compound Possible Mechanism from Breast Cancer https://doi.org/10.6084/m9.figshare.21855495. ^26^

This project contains the following reporting guidelines:

- PRISMA_2020_checklist.pdf

Figshare: Figure 1. PRISMA Flowchart. Pigmented Rice Bioactive Compound Possible Mechanism from Breast Cancer https://doi.org/10.6084/m9.figshare.21855582. ^27^

This project contains the following reporting guidelines:

- Fig 1. tiff (PRISMA flowchart)

Data are available under the terms of the Creative Commons Attribution 4.0 International license (CC-BY 4.0)
