# Supplementary material for: Benefit of Asian pigmented rice bioactive compound and its implication in breast cancer: a systematic review
Source: F1000Res. 2023 Apr 5;12:371. [Version 1] doi: 10.12688/f1000research.130329.1 (PMC10579849; doi:10.12688/f1000research.130329.1)
Supplement: Peer review history

# Reviewer report: Reviewer response for version 1

**DOI:** 10.5256/f1000research.143078.r213315

**Published:** 2023-10-16

**Authors:** Yuan Seng Wu

**Affiliations:** 1 Department of Biological Sciences, School of Medical and Life Sciences, Sunway University, Bandar Sunway, Selangor, Malaysia

The authors presented a systematic review on the Asian pigmented rice bioactive compounds and its implication in breast cancer. This manuscript is novel and has some values to the relevant community. Overall, the manuscript is acceptable in terms of writing and organisation. However, minor concerns as listed below should be addressed:

1. Title - the current title should be revised briefly to truly capture the content.
2. The authors should compare the included articles for the aspects in percentage. Like, how many articles (in percentage) presented or contained this compound or etc. Other than that, there were some missing words identified throughout the manuscript. Kindly check thoroughly. 

Are the rationale for, and objectives of, the Systematic Review clearly stated?

Yes

Is the statistical analysis and its interpretation appropriate?

Yes

Are sufficient details of the methods and analysis provided to allow replication by others?

Yes

Are the conclusions drawn adequately supported by the results presented in the review?

Yes

Reviewer Expertise:

Molecular pharmacology and therapeutics

I confirm that I have read this submission and believe that I have an appropriate level of expertise to confirm that it is of an acceptable scientific standard, however I have significant reservations, as outlined above.

## References

[1] : Tilianin: A Potential Natural Lead Molecule for New Drug Design and Development for the Treatment of Cardiovascular Disorders. *Molecules* .2022;27(3) : 10.3390/molecules27030673 10.3390/molecules27030673 35163934PMC8838974

# Reviewer report: Reviewer response for version 1

**DOI:** 10.5256/f1000research.143078.r213312

**Published:** 2023-10-16

**Authors:** Mohammed Aljunaid

**Affiliations:** 1 Faculty of Medicine, Department of Dental Medicine, Taiz University, Taiz, Taiz Governorate, Yemen

I have completed my evaluation and after reading this Systematic Review carefully I am of the opinion that this manuscript should be considered for indexing.

 Nonetheless, the authors need to resolve some minor difficulties such as the following:

 In general check the grammar.

 Methods:

1. The risk of bias needs to be added to the study or provide information on the quality assessment process.
2. Add the critical appraisal process to the review. 

In the result:

1. It is important to strike a balance between providing enough detail to support the conclusions and maintaining readability.

Are the rationale for, and objectives of, the Systematic Review clearly stated?

Yes

Is the statistical analysis and its interpretation appropriate?

Yes

Are sufficient details of the methods and analysis provided to allow replication by others?

Yes

Are the conclusions drawn adequately supported by the results presented in the review?

Yes

Reviewer Expertise:

Periodontontology, Oral Medicine, and Oral Hearbal medicine

I confirm that I have read this submission and believe that I have an appropriate level of expertise to confirm that it is of an acceptable scientific standard.

# Reviewer report: Reviewer response for version 1

**DOI:** 10.5256/f1000research.143078.r169259

**Published:** 2023-04-17

**Authors:** Ananto Ali Alhasyimi

**Affiliations:** 1 Department of Orthodontics, Faculty of Dentistry, Universitas Gadjah Mada, Yogyakarta, Special Region of Yogyakarta, Indonesia

Thank you for your work. The paper is well-researched and written, with profound insights into the bioactive compounds of Asian pigmented rice and their role in breast cancer. Best wishes to all the authors. I believe it has merit and should be considered for indexing. Novelty alone is sufficient and has a significant impact.

 In order to improve its quality I think some minor issues should be noticed:

1. Please add the evaluation of the risk of bias in the included studies.
2. This systematic review was well-designed in accordance with PRISMA guidelines. It was included in the Reporting guidelines. Please incorporate the PRISMA flowchart into the primary text.
3. Since this is a review article, it is expected that you describe some of your own premises and elucidate some novel findings from your literature studies.

Are the rationale for, and objectives of, the Systematic Review clearly stated?

Yes

Is the statistical analysis and its interpretation appropriate?

Yes

Are sufficient details of the methods and analysis provided to allow replication by others?

Yes

Are the conclusions drawn adequately supported by the results presented in the review?

Yes

Reviewer Expertise:

Orthodontic; biomaterials; biomechanic; retention in orthodontic

I confirm that I have read this submission and believe that I have an appropriate level of expertise to confirm that it is of an acceptable scientific standard.

# Reviewer report: Reviewer response for version 1

**DOI:** 10.5256/f1000research.143078.r169255

**Published:** 2023-04-13

**Authors:** Kok-Yong Chin

**Affiliations:** 1 Department of Pharmacology, Faculty of Medicine, Universiti Kebangsaan Malaysia, Kuala Lumpur, Malaysia

In this systematic review, the authors attempted to summarise the beneficial effects of Asian pigmented rice extract on breast cancer. However, the literature-gathering and evidence synthesis processes are flawed so the results and conclusion could not be judged fairly at this point. There is also no critical appraisal process, so it is hard to call this a systematic review.

- The systematic review was not constructed based on PRISMA guidelines. The authors should follow the guidelines and rewrite the manuscript for it to be considered. The authors could also refer to any published systematic review during their writing.
- Search string: Why was the bracket removed from the search string for the search in Scopus?
- Keywords: the inclusion of “cancer therapy” and “anti-metastasis” may restrict the search results. I recommend searching again without these terms.
- Setting open-access article as the inclusion criteria severely limits the search results.
- The authors need to justify setting a time frame of 10 years in the search. How would older articles not be relevant anymore?
- Why would the authors screen out items without original data (reviews, commentaries, letters etc) at the very last stage? This could be done earlier.
- Other necessary steps for deciding article inclusion are not illustrated: how many persons are making the decision, how was discrepancy resolved etc.
- There is no PRISMA flow chart to summarise the entire literature search and article identification process.
- The data extracted in the evidence table were not complete. There were no dose and treatment period data.
- The Results and Discussion sections should be revamped. The results on the benefits of pigmented rice contain information that should appear in the introduction.
- The limitation of review section discussed more on the limitations of the research field, not on the review itself.

Are the rationale for, and objectives of, the Systematic Review clearly stated?

No

Is the statistical analysis and its interpretation appropriate?

Not applicable

Are sufficient details of the methods and analysis provided to allow replication by others?

No

Are the conclusions drawn adequately supported by the results presented in the review?

No

Reviewer Expertise:

osteoporosis, osteoarthritis, evidence synthesis, vitamin E, men's health

I confirm that I have read this submission and believe that I have an appropriate level of expertise to state that I do not consider it to be of an acceptable scientific standard, for reasons outlined above.

# Response

**Published:** 2023-04-17

**Authors:** Alexander Patera Nugraha

**Affiliations:** Orthodontics, Airlangga University, Surabaya, East Java, Indonesia

Dear honorable reviewer. First of all we would like to thank your valuable comments on our article. We will try to answer your review point per point as follows:

1. This article has considered and involved critical appraisal process, such as

- Evaluation of the validity of the review

The methodological quality was assessed through the following points:

1. The question was clearly focused on breast cancer, study approach ( *in vitro* and *in vivo*), intervention and outcome measures.
2. Inclusion criteria have been indicated clearly in the article.
3. Literature search method also has been clearly specified.

- Result interpretation

The result interpretation was done according to the study approach, the type of rice and bioactive compounds role in breast cancer. This interpretation covered overall questions of this review.

- Applicability of the results of the review

This review included possible mechanism and significance of finding that will be more applicable in health or pharmaceutical practice. 

1. This article was constructed based on PRISMA guidelines and checklist.
2. This article also referred to one literature review.

Seechamnanturakit V, Karrila TT, Sontimuang C, *et al.*: The Natural Pigments in Pigmented Rice Bran and Their Relation to Human Health: A Literature Review. *KMUTNB Int. J. Appl. Sci. Technol.* 2018; **11**(1): 3–13.

 Hopefully you could understand and approve this manuscript. 

 Thank you very much.

 Sincerely Yours
